# Climate change amplified the 2009 extreme landslide event in Austria

**DOI:** 10.1007/s10584-023-03593-2

**Published:** 2023-08-26

**Authors:** Aditya N. Mishra, Douglas Maraun, Raphael Knevels, Heimo Truhetz, Alexander Brenning, Herwig Proske

**Affiliations:** 1 Wegener Centre for Climate and Global Change, University of Graz, Graz, Austria; 2 Friedrich Schiller University Jena, Department of Geography, Jena, Germany; 3 Remote Sensing and Geoinformation Department, JOANNEUM RESEARCH, Graz, Austria

**Keywords:** Attribution, Landslide, Extreme rainfall, Climate change

## Abstract

Landslides are an important natural hazard in mountainous regions. Given the triggering and preconditioning by meteorological conditions, it is known that landslide risk may change in a warming climate, but whether climate change has already affected individual landslide events is still an open question, partly owing to landslide data limitations and methodological challenges in climate impact attribution. Here, we demonstrate the substantial influence of anthropogenic climate change on a severe event in the southeastern Alpine forelands with some estimated 952 individual landslides in June 2009. Our study is based on conditional event attribution complemented by an assessment of changes in atmospheric circulation. Using this approach, we simulate the meteorological event under observed and a range of counterfactual conditions of no climate change and explicitly predict the landslide occurrence probability for these conditions. We find that up to 10%, i.e., 95 landslides, can be attributed to climate change.

## Introduction

Landslides are an important threat to population and infrastructure in mountainous regions across the globe (Yang et al. 2015), and one of the major natural hazards in the European Alpine forelands (Jaedicke et al. 2014). The susceptibility of a region to landslides depends on the topographical, geomorphological, geological, and land use and land cover (LULC) conditions (Schweigl and Hervás 2009). In the Alpine forelands, landslides are mainly triggered by persistent spells of rain, intense short-duration rain showers, and rapid snow melt, preconditioned by high soil moisture (Crozier 2010; Gariano and Guzzetti 2016; Mostbauer et al. 2018; Maraun et al. 2022).

Human-induced climate change has contributed to the changes in the drivers of landslide occurrence (Gariano and Guzzetti 2016). In particular, the frequency and intensity of heavy rainfall events have increased at the global scale over the majority of land regions (Seneviratne et al. 2021; Kiktev et al. 2007; Min et al. 2011; IPCC 2021). Heavy summertime rainfall, though with uncertainties, is increasing over the Greater Alpine Region (GAR) as well (Rajczak and Schär 2017). Rainfall intensities over the GAR associated with Mediterranean cyclones also increase with a warming climate (Volosciuk et al. 2016; Nissen et al. 2013; Messmer et al. 2017; Maraun et al. 2022), along with more severe impacts (Stuart-Smith et al. 2021; Mitchell 2021).

The question of whether individual landslides have already been affected by climate change is an important scientific question, that may be relevant for addressing loss and damage issues (Lusk 2017) and aid in communicating how much climate change already now affects ecosystems and societies (Begum et al. 2022). The actual influence of climate change on particular observed landslide events has not yet been quantified, i.e., a full climate change event attribution study for landslides has not been conducted. This is because, firstly, in the case of landslides, inventories are often not well-dated such that linking the landslides to a particular meteorological event is impossible (Van Westen et al. 2006). Secondly, even though attribution of meteorological extreme events to climate change has a long tradition (Stott et al. 2004; Van Oldenborgh et al. 2017; Philip et al. 2018; Wolski et al. 2014; Perkins-Kirkpatrick et al. 2022; Trenberth et al. 2015), based on a range of methodologies (Seneviratne et al. 2021; Stott et al. 2016; Shepherd 2016), the attribution of impacts to climate change is a much more recent and intricate endeavour (Begum et al. 2022; Perkins-Kirkpatrick et al. 2022). Rapid attribution studies on recent events, such as the South African flooding-landslide event in 2022, only address the attribution question through the meteorological perspective (Singh et al. 2022). In particular, meteorological attribution statements cannot be naively translated to impacts because of the often complex response of the impact systems to meteorological drivers (Perkins-Kirkpatrick et al. 2022). This is particularly true for landslides, as rainfall and soil moisture changes are known to have an opposing influence on the landslide occurrence response (Maraun et al. 2022; Knevels et al. 2023).

**Fig. 1:**
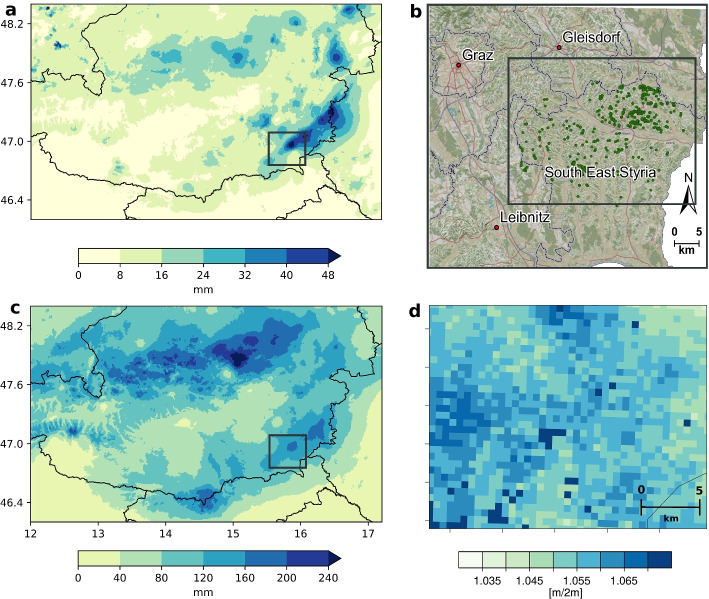
**The rainfall and landslide event 2009** **a** 3-hourly maximum rainfall between 22 and 26 June 2009. **b** Map of 518 recorded landslides during the 2009 event (in green dots). Data: GISCO NUTS 2013, OpenStreetMap, INCA. **b** 5-day aggregate rainfall ending between 22 and 26 June 2009 in the INCA (Integrated Nowcasting through Comprehensive Analysis) data set. (**d**) Hindcast simulation of maximum 2-m-integrated soil moisture in the target region on the day prior to the beginning of the corresponding 5-day rainfall aggregation period (see Section 2 for details. Target region for soil moisture, marked by a black box in (**a**), (**b**), and (**c**))

Thus, in order to quantify the influence of climate change on a particular landslide event, one must conduct a full attribution study whereby the influence of climate change on rainfall and landslide events is assessed discretely. Here, we study a rainfall-landslide event that happened during June 22–26, 2009, in central Europe. This event is especially suitable for event attribution as it has a well-dated landslide inventory along with good meteorological observations which help in drawing robust attribution statements. The landslide event was triggered by a slow-moving cut-off low that brought warm moist air to central Europe leading to incessant spells of heavy rainfall (Hornich and Adelwöhrer 2010). The Eastern Alps and its adjoining foothills were particularly affected (Haiden 2009), as some weather stations recorded high-intensity rainfall spells of 50 mm within 3 h (Fig. 1a). The persisting rainfall event led to catastrophic scenes with multiple instances of flooding in the northeastern Alpine forelands (Godina and Müller 2009), and several hundred landslides were reported in the Feldbach area of the southeastern Austrian state of Styria (Hornich and Adelwöhrer 2010) (Fig. 1b; not all counted events should be classified as landslides, we estimate a number of 952; see Sections 2.2 and 2.5 for details). A state of emergency was issued in Feldbach (Möseneder 2009), and the estimated reparation costs for the state of Styria surpassed 13.4 million euros, excluding damages paid privately or by insurance companies (Hornich and Adelwöhrer 2010). Besides the triggering rainfall, high soil moisture may also have contributed to the number of landslides as the preceding winter was particularly snowy (Hornich and Adelwöhrer 2010) (Fig. 1d).

We chose to implement a conditional attribution approach (Trenberth et al. 2015), over the traditional probabilistic attribution approach (Stott et al. 2016) in this study. While García-Portela and Maraun (2023) discuss differences between, and limitations of, the two approaches, Lloyd and Shepherd (2020) argue that the conditional approach is particularly suitable for impact attribution. In our case, the use of conditional attribution is particularly useful because it first allows us to simulate extreme rainfall using a very high-resolution climate model to resolve local processes and topography. Second, the separation of dynamical and thermodynamic changes avoids large-scale circulation biases affecting the local simulations and thus allows for directly linking simulated hydro-meteorological conditions to the local landslide event (Maraun et al. 2022). Third, since we only have a single event with landslide data, we cannot model landslides for long-term climate model output, as in the traditional approach. The conditional attribution takes the atmospheric circulation underlying the event as given. Therefore, to consider the effect of changes in the atmospheric circulation (Otto et al. 2016), we complement the conditional approach with a separate literature-based assessment of changes in cut-off low occurrence frequency from the state-of-the-art CMIP5 and CMIP6 GCMs.

## Approach and methods

### Approach

For the conditional attribution, we use a model-based event storyline approach (Shepherd et al. 2018; Sillmann et al. 2021; Lloyd and Shepherd 2020), specifically designed for the assessment of landslides in a changing climate (Maraun et al. 2022). First, we simulate the meteorological event as it happened in 2009 (actual conditions) using the CCLM regional climate model (RCM) (Böhm et al. 2006; Rockel et al. 2008) at a convection-permitting resolution over the Eastern Alps. Observed boundary conditions are taken from reanalysis data. We then simulate the event again under counterfactual conditions representing a world without anthropogenic climate change, i.e., one-degree cooler compared to the actual conditions. To this end, we modify the 3-dimensional boundary conditions (temperature, humidity, and sea level pressure) by a range of plausible changes simulated in general circulation models (GCMs) for similar events, representing a one-degree cooler world. The simulations, both for actual and counterfactual conditions, also cover the preceding winter to represent soil moisture changes resulting from a different snow cover, spring precipitation, and evapotranspiration (see Section 2.3 for details).

**Fig. 2:**
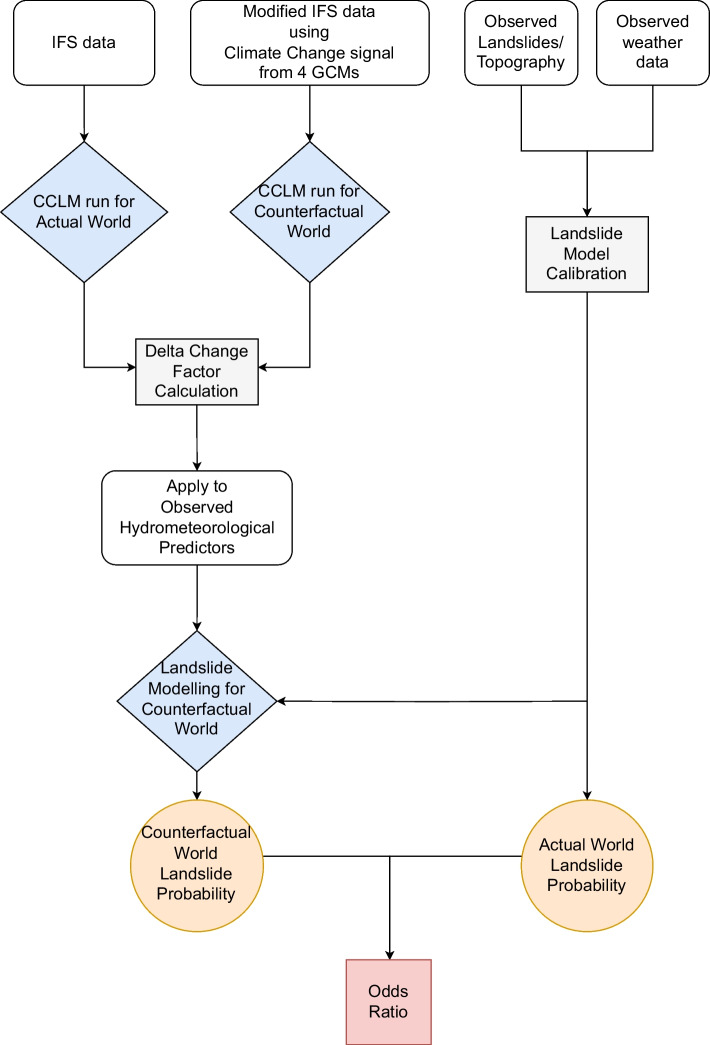
Flow chart description of the approach

We represent uncertainties in the climate change response of the meteorological event by simulating four different storylines, derived from four different GCMs, spanning a wide range of plausible regional climate responses to anthropogenic climate change. Thereafter, we assess landslide occurrence probability for both, actual and counterfactual, worlds using a nonlinear statistical regression model (Knevels et al. 2020; Maraun et al. 2022; Knevels et al. 2023). The spatial and magnitudinal extent (geometry) of the landslides is not assessed in this study (this is because different federal state institutions were involved in recording the landslides in the field, and unfortunately, without a consistent approach, only very few landslides were mapped in their full geometry or spatial extent). For a given 10 m × 10 m location, the model predicts the landslide occurrence probability from time-invariant topographical, geological, and geomorphological, LULC conditions as well as meteorological predictors representing aggregated rainfall and highest rainfall intensities during the event, and soil moisture conditions prior to the event (see Section 2.5 for details). Rainfall and soil-moisture response are taken in from the high-resolution climate simulations while the LULC is deliberately taken unchanged between the actual and the counterfactual worlds in our experiments—to isolate the effect of anthropogenic warming on landslides. We use the term ‘response’ for denoting a change in the predictor in the counterfactual world, compared to the actual. Our approach is illustrated through a flow chart in Fig. 2.

In previous studies, we identified the following best meteorological predictors for landslide occurrence at a given location (Knevels et al. 2020; Maraun et al. 2022): 3-hourly maximum rainfall on the occurrence day of a landslide, representing short-duration high-intensity downpours; 5-day aggregate rainfall prior to the occurrence of a landslide, representing the persistent rain; and 2-m-integrated soil moisture prior to the rainfall aggregation period representing the preconditioning.

### Data

The data used here are the same as those used by Maraun et al. (2022). The landslide model is calibrated using data from two different landslide events that happened in the region, in 2009 and 2014. Precipitation data used to derive the present-day meteorological predictors for the statistical landslide model are from the INCA (Integrated Nowcasting through Comprehensive Analysis) nowcasting system (Haiden et al. 2011) of the ZAMG (Zentralanstalt für Meteorologie und Geodynamik). Due to the lack of quality soil moisture observation data available, we use simulated soil moisture data (representing the 2009 and 2014 events) as a predictor for the statistical model (Maraun et al. 2022) derived using the HRLDAS (high-resolution land data assimilation system) (Chen et al. 2007). The model itself is initiated using the ERA-Interim (ECMWF ReAnalysis-Interim) data (Dee et al. 2011). The simulations are performed on a 1 km × 1 km grid at an hourly resolution within the 2004–2014 period. Soil texture types and land surface parameters are obtained from the WRF Noah-LSM (Weather Research and Forecasting-Noah land surface model). For details on forcing data, please refer to Maraun et al. (2022), and for topographical predictors and further details, please refer to Knevels et al. (2020). All predictors are interpolated to a 10 m $\times$ 10 m resolution, and each landslide is represented by a point.

We calibrate the statistical landslide model on two different (but geographically close) events to constrain the relationship between soil moisture and landslide occurrence (Maraun et al. 2022). In each single event, soil moisture variations across space were small, essentially hiding any predictive power of soil moisture for landslide occurrence. But as the two events happened under different soil moisture conditions, the joint calibration to both events enabled to identify the soil moisture influence on landslide occurrence. Including soil moisture was essential to make the model fit for climate projections. Moreover, in Knevels et al. (2020), we investigated different calibration settings and found the combined data sets to deliver best and physically meaningful results in terms of model performances and predictor–predictand relationships (model GAM-Co, in that paper). These two rainfall events resulted in hundreds of individual landslides, but for calibration purposes, we focused on earth and debris slides with possible transitions to complex slide flows (Cruden and Varnes 1996), and we used only a selected number of landslides based on expert judgement and minimum size. A total of 626 landslides were taken, 487 of which were from 2009 and 139 from the 2014 event. This data are recorded and provided by the Institute of Military Geoinformation, the Geological Survey of Austria (2009), and the Styrian Government (2014) (Knevels et al. 2020; Kautz 2010).

For our analysis, however, we only take 518 out of them. This is for two reasons: first, many of the reported events were from a broader class of events such as cracks in streets or general erosive events. We have filtered these out because they are of different landslide types and underlying physical processes. Second, the high-quality inventories available to us did not cover the full Feldbach district but rather about 40% of the total area (Knevels et al. 2020). Also, we used a spatiotemporal sampling design to position absent observations, and areas where landslides are not expected to occur (trivial areas) such as flat terrains in valley bottoms were excluded.

### Meteorological simulations

The meteorological simulations are conducted using the ‘Consortium for Small-scale Modelling’ (COSMO) model in climate mode or CCLM (RCM) (Böhm et al. 2006; Rockel et al. 2008) over the domain 44.5 N–49.1 N and 10.7 E–19.8 E which roughly coincides with the eastern Alps. ‘Eastern Alps’ in our context is an area defined for our modelling purpose and consists of central-eastern Austria, adjoining parts of Germany, Italy, Slovenia, and Hungary. The simulations are performed in convection-permitting mode at 3 km $\times$ 3 km grid space. A list of model parameterization schemes used is available in Supplementary Table 1 of Maraun et al. (2022). Soil moisture is also explicitly simulated by the land-surface component of the model. Boundary conditions for simulating the rainfall event under present-day conditions are derived from the Integrated Forecasting System (IFS) of the ECMWF (Bechtold et al. 2008). The modelling experiment is backed by a spin-up simulation period of 9 months starting 01-10-2008 00:00 UTC until 20-06-2009 00:00 UTC. The spin-up simulation helps to create a balanced soil moisture field that would accommodate the preceding winter and spring of 2008/09. Thereafter, a ten-member ensemble simulation is performed for the period 20-06-2009 00:00 UTC until 28-06-2009 00:00 UTC. The initial conditions for the ensemble members are perturbed by staggering the starting time of the simulations, from 20-06-2009 00:00 UTC at three-hourly intervals backward in time.

To generate the counterfactual storylines, we alter the RCM boundary conditions in the following way such that it thermodynamically represents a one-degree cooler world: Two reference periods are selected to calculate the climate change signal: a historic period from 1975 to 2004 and a future period from 2071 to 2100. The reason behind choosing the aforementioned periods for calculating a change signal, for a counterfactual cooler world, is a better signal-to-noise ratio when sampling the extreme events in the present and future worlds. We identify 4 CMIP5 GCMs at the RCP8.5 (highest Representative Concentration Pathways) scenario that represent a wide range of uncertainties and also have data available at multiple atmospheric levels in the selected time periods—this is critical as we need 3-D climatic changes. The calculation of the climate change signal for the 9-month spin-up period (01-10-2008 to 20-06-2009) is done by subtracting the 3-D mean temperature, relative humidity, and mean sea-level pressure (MSLP) of the historic period from the future period. However, for the period 20-06-2009 to 28-06-2009 (days surrounding the event), we require boundary conditions that represent the climate conditions similar to that of 2009-type events, i.e., extreme rainfall events. To this end, for this period, we calculate the empirical $99^{th}$ percentile of 3-day aggregated grid-box rainfall in the region 13.7 E–17.5 E and 46 N–48 N (East Austria) for the June-July-August (JJA) months, separately for the above-mentioned 30-year periods in each considered GCM. Thereafter, we calculate the average vertical profiles of all summer days with events wetter than $99^{th}$ percentile of rainfall over the RCM domain of the 3-D temperature and relative humidity fields from the surface to the lower stratosphere (35 hPa) as well as MSLP. The vertical profiles and the MSLP of the historic period are subtracted from the future period; this gives a 3-D climate change effect on temperature, relative humidity, and MSLP, representative of such extreme events.

The 3-D profiles calculated above are linearly scaled to one-degree cooling from the present. This is done by rescaling the regional changes in the 3-D profiles with the ratio between the global mean surface temperature change ($\Delta T_{cpast}$) for a one-degree cooler world compared to the present and the simulated global mean surface temperature change for the chosen GCM simulation (1975–2004 to 2071–2100 at RCP8.5). Finally, these average changes are to be applied to the IFS data (driving data) that now represents modified (counterfactual) lateral boundary conditions. These modified IFS data are used in the RCM model to perform the counterfactual simulations. To this end, the surface pressure is modified with the changes in sea level pressure taking into account the local orography of the IFS, instantaneously changing the IFS pressure levels. To maintain physical consistency and satisfy the hydrostatic equation, temperature and relative humidity are adjusted according to the new vertical extensions of the pressure levels. Thereafter, the 3-D profiles are added, and the specific humidity is calculated again from the modified temperature, relative humidity, and pressure fields using the Clausius-Clapeyron equation.

The selected GCMs of IPSL-CM5A-MR (negative change), HadGEM2-CC (negative change), GFDL-ESM2m (no change), and MIROC-ESM (strong positive change) represent a broad range of precipitation changes from the CMIP5 ensemble.

### Delta change approach for the predictors

To determine the accuracy of our modelling setup, we compare the observed INCA rainfall with the simulated rainfall under actual conditions. We find fairly good agreement between the intensity and spatial distribution of rainfall across both sets of data, but they are not identical and have small location biases (Maraun and Widmann 2015). Simulations for the counterfactual one-degree cooler scenario also feature a slight spatial shift compared to the present-day simulated event. We also notice similar biases between soil moisture simulations and reference simulations. By climate modelling standards, these shifts are small compared to the domain size and our simulations do fairly well in representing the rainfall event in its extent and intensity. However, even small shifts may cause substantial biases in the landslide modelling, which requires very precise localization of landslide-inducing rainfall patterns.

To navigate this issue, a delta change approach is used whereby we do not directly consider the simulated hydrometeorological predictor fields but rather use them to calculate change factors that are subsequently applied to the observed hydrometeorological predictor fields (Maraun et al. 2022). In a standard change factor approach, long-term differences between simulated future and present temporal means would be calculated for a specific grid box, and these differences would then be added to present-day observations. In the case of precipitation, ratios are considered instead of differences. But, since we are specifically examining a single event of a few days in length, it is not reasonable to average over time. Therefore, we perform spatial averaging over a region where the climate change signal can be assumed to be relatively constant. For the rainfall fields, we apply area-averaging over the heavy-rainfall domain spanning from 13.82 E to 17.31 E and 46.16 N to 48.01 N. This domain is chosen to account for shifts in local rainfall patterns while excluding the higher mountains of the Alps to prevent biases in the factor calculation. For soil moisture, we perform averaging across the actual target domain to avoid the influence of geological variations outside of that specific domain on the climate change signal. The delta change factors are derived separately for all predictors, i.e., 3-hourly maximum, 5-day aggregate rainfall, and soil moisture. By calculating and applying the change factors to individual predictor fields, we keep the difference in climate change signals for different predictors. To average out the local internal variability, the factors are calculated on all permutations of ten present-day and ten one-degree cooler ensemble members for the rainfall predictors. This delta change approach aids in maintaining the spatial accuracy of the event while intensity changes are resolved by the convection-permitting regional model.

### Landslide modelling

For the estimation of landslide occurrence probability, a semi-parametric generalised additive model is used (Knevels et al. 2020; Hastie and Tibshirani 2017; Wood 2006) that links $m$ predictors $x_{i}, i=1...m$ via transformation functions $f_{i}(.)$ and a link function $g(.)$ to the conditional expected value $E(.)$ of the  response. Here, the logit link function is used as the default link function for a binomial distribution. The logit of landslide occurrence probability, g(E(Y)), within a grid cell, is then modelled additively as follows:

$$\begin{aligned} g(E(Y))=\beta _{0}+f_{1}(x_{1})+..+f_{m}(x_{m}). \end{aligned}$$

Model setup and selection of predictors are based on Knevels et al. (2020) but have been extended for climate change applications (Maraun et al. 2022). The model treats ‘shallow, rapid’ and ‘rapid, deep’ landslide events jointly (Sidle and Ochiai 2006). Since landslide inventories are often sparse, we evaluated the combination of both events in terms of spatiotemporal cross-validation and predictor–predictand relationships (Knevels et al. 2020). We discovered that a combined-event model showed significantly higher performance estimates (i.e., $\Delta$ median AUROC values of 0.03$-$0.06, Table A6 in Knevels et al. (2020)). Moreover, we found that the predictor–predictand relationships are physically more plausible and less variable (Fig. 6 in Knevels et al. (2020)) compared to single-event models. To account for changes in preconditioning soil moisture along with climate change, we included soil moisture as an additional predictor for landslide occurrence. For details, please refer to Knevels et al. (2020) and Maraun et al. (2022).

Landslides are rare events, normally having occurrence frequencies that are low, which means, dozens to thousands of times smaller than the number of non-events. The rareness of landslide events typically results in an overestimation of landslide occurrence probabilities (King and Zeng 2001). Because odds ratios are invariant to such biases, we express changes in landslide occurrence probabilities as odds ratios (Szumilas 2010) (Fig. 4 a–d). Odds associated with a probability $p$ are defined as $O=p/(1-p)$. Odds ratios of counterfactual past and present landslide occurrence are then defined as the ratio between past and present odds:

$$\begin{aligned} OR_{past/pres}=\frac{O_{past}}{O_{pres}}=\frac{p_{past}(1-p_{pres})}{p_{pres}(1-p_{past})} \end{aligned}$$

Our landslide model is designed to predict odds ratios conditional on different environmental predictors, but giving the influence of climate change on actual landslide occurrence probabilities—or even absolute landslide numbers—would aid the communication of our results. Unfortunately, landslides are rare events, inducing a bias in the logistic regression using a specified sampling ratio that does not affect odds ratios but actual probabilities. To eliminate this bias, we apply a rare event correction of the intercept for the predictions (please see Eq. (7) in King and Zeng (2001)). The correction factor takes into account the actual low fraction of landslides in the sampling area ( 0.01%) in relation to the 1:5 sampling strategy used for model fitting (Knevels et al. 2020), thereby considering the sampling design. Also, the presence–absence ratio of 1:5 was selected to account for random variability in the underlying spatio-temporal sampling design (Knevels et al. 2020). In our case, the correction is 7.651 on the logit scale. We transform the biased landslide occurrence probabilities for all individual cells into logit values, subtract the correction factor, and transform back to obtain corrected estimates of landslide occurrence probabilities.

Using this correction factor, we predict a total number of 1363 landslides over the sub-region of the Feldbach region used for calibrating the statistical model (the landslide-free masks in Figs. 2B and 3B of Knevels et al. (2020)). Recall that we have considered 518 landslide events across this sub-region for the model calibration. This discrepancy between the predicted and observed landslide numbers is because the landslide model is not calibrated to reproduce (as predictions) the number of events observed in a data set or region. Thus, we can only interpret relative changes in landslide numbers, not absolute changes. To provide approximate absolute numbers for better communicating the influence of climate change on the event, we therefore introduce an adjustment factor by simply rescaling the predicted landslide number to match the observed number over the considered sub-region. In our case, this adjustment factor is 518/1363 = 0.379. Thereafter, we apply this factor to landslide number predictions for the entire Feldbach region in the actual and counterfactual climates. For the actual event as it happened, we predict 2512 landslides for the entire Feldbach region, i.e., 952 landslides after the adjustment. For the counterfactual simulations, we calculate landslide numbers equivalently.

The use of the adjustment factor is necessary for the calculation of the estimated absolute landslide number. However, the methodology leading up to the adjustment factor has some limitations. These include ignoring landslide volume and size; thus, a large landslide and a small landslide have the same single-point geometry. Additionally, landslides in the Feldbach area might be overseen during recording from the federal state institutes. Also, the established landslide-free masks and the taken assumptions for their delineation might have been too restrictive (Knevels et al. 2020). This may have resulted in masked ‘unseen’ areas for absence positioning, which were actually ‘seen’ (this affects $\tau$, i.e., the  0.01% of the cells that were envisaged to represent landslide initiation, and ultimately may lead to a smaller correction factor (Eq. (7) in King and Zeng (2001))).

## Results and discussion

### Attribution of the meteorological event

**Fig. 3:**
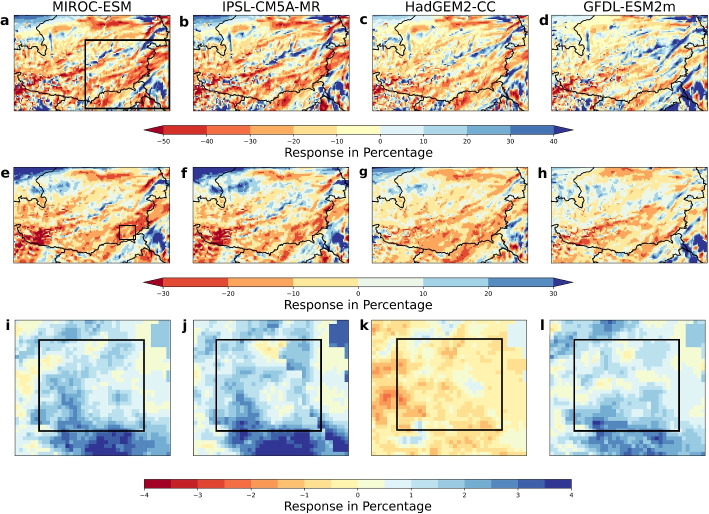
**Percentage response in rainfall and soil moisture compared to actual conditions**. (**a–d**) 3-hourly maximum rainfall. (**e-h**) 5-day aggregate rainfall. (**i-l**) 2 m-integrated soil moisture in the area marked with a black rectangle in (**e**) (target region for soil moisture response calculation). The rectangle marked in black in (**a**) represents the domain used to calculate the delta change factors for the rainfall predictors

Our storyline simulations covering the entire eastern Alpine region show that the 3-hourly maximum rainfall response is up to 50% lesser compared to the actual conditions in the four selected GCMs (Fig. 3a–d). Whereas, the 5-day aggregate rainfall response is also up to 30% lesser compared to the actual conditions (Fig. 3e–h). 2 m-integrated soil moisture response is wetter by up to 4% (Fig. 3i, j, l), barring one GCM where the response is 2% drier compared to the actual conditions (Fig. 3k).

Although in this study we focus on the landslide event, the reduction in both 3-hourly maximum and 5-day aggregate rainfall strongly indicates that also the flooding events in the northern Austrian states of Upper and Lower Austria have already been amplified by anthropogenic climate change.

**Fig. 4:**
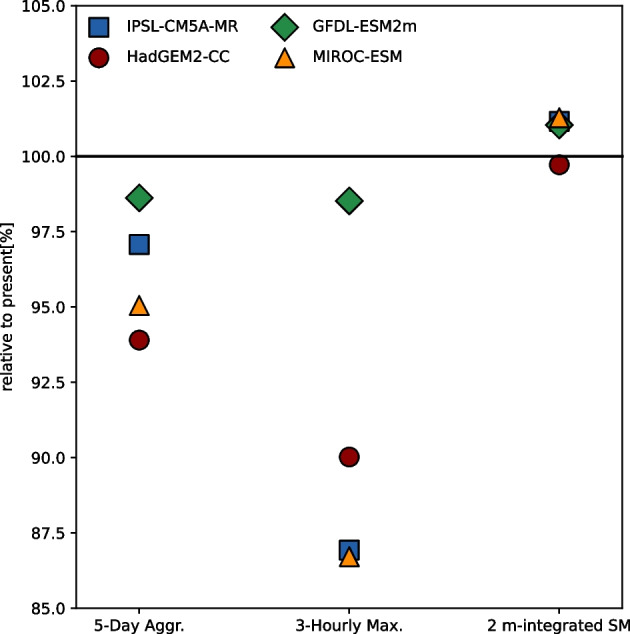
Storylines of area-mean rainfall and soil moisture changes

We develop a delta change approach (see Section 3.3) to calculate area average changes for each of our three predictors and apply these changes to the observed predictor fields. These area mean changes for the four meteorological storylines are shown in Fig. 4, and the main features of these storylines are presented in Table 1.

**Table 1:** Hydrometeorological storylines

Model	5-day rain	3-h max rain	Soil moisture	Storyline description
MIROC-ESM	Strong decrease	Strong decrease	Increase	Much lighter rain, moist soil
IPSL-CM5A-MR	Decrease	Strong decrease	Increase	Lighter rain, moist soil
HadGEM2-CC	Strong decrease	Decrease	Marginal change	Lighter rain
GFDL-ESM2m	Marginal change	Marginal change	Increase	Moist soil

Soil moisture values refer to the day prior to the 5-day aggregation period

The following storyline results for the three predictors are based on area-averaged changes in the target region for rainfall and soil moisture and thus account for the small location biases in the rainfall field:

In the counterfactual, one-degree cooler world, the 3-hourly maximum rainfall intensities are lower by approximately up to twice the Clausius-Clapeyron rate in three storylines (“much lighter rain, moist soil”, “lighter rain, moist soil”, and “lighter rain”), whereas the reduction in intensity is marginal in one storyline (“moist soil”). Super Clausius-Clapeyron scaling of short-duration extreme rainfall has been linked to feedbacks associated with latent heat release in convective updrafts (Lenderink et al. 2017).

The response in 5-day aggregate rainfall is a decrease in intensity of up to 7% in two storylines (“much lighter rain, moist soil” and “lighter rain”) compared to the actual world—consistent with the Clausius-Clapeyron rate. The response is weaker in the other two storylines at 2–3%, likely owing to increased atmospheric stability (Fowler et al. 2021).

Soil moisture responses are relatively small compared to the responses in the rainfall predictors. The soil is about 1% moister in the counterfactual world in three storylines (“much lighter rain, moist soil”, “lighter rain, moist soil”, and “moist soil”), while a marginal drying of 0.3% in one storyline (“lighter rain”).

The conditional event attribution addresses the question ‘How much has climate change affected the event, given the cut-off low responsible for the persistent heavy rainfall?’ To arrive at a full attribution statement, we further address the question of how climate change might have already altered the occurrence of cut-off lows. So far, no studies about changes in cut-off lows due to anthropogenic climate change exist, and analysing model projections was beyond the scope of this study. Nevertheless, we wanted to look into the forced changes and cut-off lows often occur in conjunction with atmospheric blocking (Nieto et al. 2007). Using changes in atmospheric blocking frequency as a proxy (Maraun et al. 2022), CMIP5 and CMIP6 models suggest that climate change has slightly reduced the frequency of European summer blocking by about 4% per degree of global mean warming (Davini and d’Andrea 2020). Thus, while climate change has only slightly affected the occurrence probability of the event, the rainfall intensification associated with the event is substantially stronger.

### Attribution of the landslide event

**Fig. 5:**
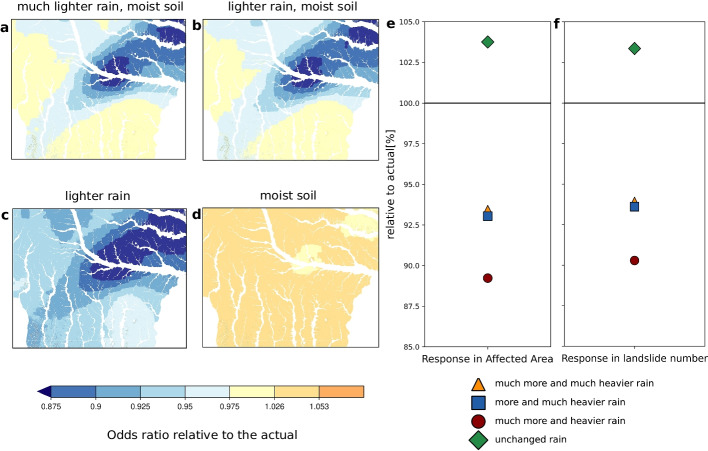
**Maps of landslide storylines and storylines of the affected area in the Feldbach region**. (**a–d**) Odds ratios of landslide occurrence probability during the 2009 event happening in a one-degree cooler climate relative to the present climate in the four given storylines. (**e**) Response in the area affected by a high landslide occurrence probability and (**f**) response in the number of landslides with a high occurrence probability of at least 68% within a 10 m $\times$ 10 m cell (corresponding to the 95th percentile of landslide occurrence probability across all cells in the Feldbach region in present climate) during the 2009 event happening in a one degree cooler climate. The horizontal black line indicates the actual-day reference

Compared to the actual world, local landslide occurrence odds are up to 16% lower in the counterfactual world in the “lighter rain” storyline (Fig. 5c) due to the substantially lower 5-day aggregate rainfall activity and no change in soil moisture conditions. In the other (“much lighter rain, moist soil” and “lighter rain, moist soil”) storylines, the landslide occurrence odds are lowered by as much as 14% (Fig. 5a, b).

The area at high risk of landslide occurrence (see Fig. 5 for definition) is substantially lower by about 11% in the “lighter rain” storyline and up to 7% lower in the “much lighter rain, moist soil” and “lighter rain, moist soil” storylines, compared to the actual world. Only in the “moist soil” storyline, the area at high risk is larger by approximately 3.5% (Fig. 5e) because of the weak rainfall response and slightly wetter soil in the counterfactual world.

To further illustrate the impact of climate change, we assess how many of the actual landslides occurring in the region can be attributed to climate change. We estimate that some 952 landslides occurred during the 2009 event (for a discussion of this estimate, see Sections 2.2 and 2.5). In the counterfactual world, this number dropped by as much as 10%, i.e., approximately 95 landslides in the “lighter rain” storyline. In storylines “much lighter rain, moist soil” and “lighter rain, moist soil”, this number is lowered by up to 6.5%, while a slight rise in the number of about 3% is also possible in the “moist soil” storyline (Fig. 5f).

### Conclusion and discussion

Attribution studies often examine the human influence on an extreme rainfall event only through the meteorological perspective. However, the link between a meteorological event and its impact might be complex and nonlinear, such that attribution statements cannot be directly transferred to the impact (Perkins-Kirkpatrick et al. 2022). In this study, for the first time, we explicitly demonstrate the impact anthropogenic climate change already had on a severe landslide event, through full impact attribution analysis. Using the storyline approach, we are able to fully exploit a well-dated landslide inventory and separate the influence of thermodynamic response from the large-scale circulation. Additionally, we demonstrate the opposing influence of rainfall and soil moisture changes on the event. We show that without climate change, the 2009 severe landslide event would have seen some 857 landslides, which is 95 landslides less than the estimated number of 952. Therefore, 10% of the landslides that happened in the 2009 event can be directly attributed to climate change. The substantial increase in the number of landslides by anthropogenic climate change is only weakly moderated by a slight decrease in the occurrence probability of the underlying cut-off low. These results provide further evidence of the dramatic effects climate change already has on our environment and infrastructure (IPCC 2022).

Changes in landslide occurrence with climate change depend strongly on season, region, and elevation (Gariano and Guzzetti 2016; Stoffel et al. 2014; Paranunzio et al. 2019), and uncertainties in projections are high because of a lack of well-dated landslide observations and limitations in the climate-landslide modelling chain (Gariano and Guzzetti 2016; Maraun et al. 2022). In this context, our results and those of Maraun et al. (2022) demonstrate how reductions in soil moisture counteract rainfall increases. This discussion also holds relevance for similar types of mass movements, such as debris flow, whereby more voluminous landslide events in the future are predicted (Kaitna et al. 2023).

Our findings not only underline the relevance of impact attribution, i.e., including a landslide model in the assessment, but also expose the uncertainties therein. While the responses of the individual hydro-meteorological variables have substantial uncertainty, they are still very certain about the direction of change. The opposing influence of these variables, however, causes considerable uncertainty about the impact response (Knevels et al. 2023). As a result, we cannot fully rule out that climate change has even reduced the number of landslides. This uncertainty in impact response is only brought to light due to the full impact attribution.

Impact attribution is hampered by the scale mismatch between the large-scale climate model ensemble simulations and the often small scale of the impact. Our study highlights the power of event storyline approaches to overcome this gap. It allows us to separate the local scale from large-scale simulations and thus choose optimal modelling strategies for both scales. Simulating only a single event further allows us to use a high-resolution climate model in tandem with a landslide model to explicitly simulate the impact. The approach used in the study is transferable to events with a good available inventory. Hence, the approach proposed in this study—conditional event attribution down to the impact, complemented by an assessment of large-scale circulation changes—provides a powerful avenue to advance impact attribution (Perkins-Kirkpatrick et al. 2022).

## Data Availability

IFS boundary conditions for the CCLM RCM can be obtained from the ECMWF https://www.ecmwf.int/en/forecasts/datasets (cycle35r2). ERA5 and ERA-Interim reanalysis data from ECMWF are available from https://www.ecmwf.int/en/forecasts/datasets/browse-reanalysis-datasets. Data from the chosen CMIP5 GCM simulations can be downloaded from the Climate and Environmental Retrieval and Archive (CERA) Database (https://cera-www.dkrz.de/WDCC/ui/cerasearch). Rainfall data from the Integrated Nowcasting through Comprehensive Analysis (INCA) System is available from the Austrian Meteorological Service (https://data.hub.zamg.ac.at). Landslide data can be requested from the State of Styria (raimund.adelwoehrer@stmk.gv.at), Geological Survey (GBA, arben.kociu@geolba.ac.at), and the Institute of Military Geoinformation (IMG, helene.kautz@bmlv.gv.at). All topographical predictors are publicly available from GIS-Steiermark (http://www.gis.steiermark.at, German only).
